# Why we need easy access to all data from all clinical trials and how to accomplish it

**DOI:** 10.1186/1745-6215-12-249

**Published:** 2011-11-23

**Authors:** Peter C Gøtzsche

**Affiliations:** 1 Nordic Cochrane Centre, Rigshospitalet, University of Copenhagen, Blegdamsvej 9, 2100 Copenhagen Ø, Denmark

## Abstract

International calls for registering all trials involving humans and for sharing the results, and sometimes also the raw data and the trial protocols, have increased in recent years. Such calls have come, for example, from the Organization for Economic Cooperation and Development (OECD), the World Health Organization (WHO), the US National Institutes of Heath, the US Congress, the European Commission, the European ombudsman, journal editors, The Cochrane Collaboration, and several funders, for example the UK Medical Research Council, the Wellcome Trust, the Bill and Melinda Gates Foundation and the Hewlett Foundation.

Calls for data sharing have mostly been restricted to publicly-funded research, but I argue that the distinction between publicly-funded and industry-funded research is an artificial and irrelevant one, as the interests of the patients must override commercial interests.

I also argue why it is a moral imperative to render all results from all trials involving humans, also healthy volunteers, publicly available. Respect for trial participants who often run a personal and unknown risk by participating in trials requires that they - and therefore also the society at large that they represent - be seen as the ultimate owners of trial data.

Data sharing would lead to tremendous benefits for patients, progress in science, and rational use of healthcare resources based on evidence we can trust. The harmful consequences are minor compared to the benefits. It has been amply documented that the current situation, with selective reporting of favorable research and biased data analyses being the norm rather than the exception, is harmful to patients and has led to the death of tens of thousands of patients that could have been avoided.

National and supranational legislation is needed to make data sharing happen as guidelines and other voluntary agreements do not work. I propose the contents of such legislation and of appropriate sanctions to hold accountable those who refuse to share their data.

## Background

International calls for registering all trials involving humans and for sharing the results - and sometimes also the raw data and the trial protocols - have increased in recent years. Calls for such data sharing have mostly been restricted to publicly-funded research, but I argue here that the distinction between publicly-funded and industry-funded research is an artificial and irrelevant one, as the interests of the patients must override commercial interests. The main focus of this paper is therefore drug trials.

I also argue why data sharing would lead to tremendous benefits for patients, progress in science, and rational use of healthcare resources based on evidence we can trust, and that the harmful consequences are minor compared to the benefits.

My paper aims at convincing those who have doubts about whether we should share our research data. It is less focused on practical or legal difficulties, which can always be resolved if there is a willingness to resolve them, but I do suggest the introduction of a new law about data sharing and sanctions in case the law is violated.

The fundamental problem is that, with rare exceptions, we do not know what the true benefits and harms of our interventions are. This may seem counterintuitive, given the existence of hundreds of thousands of randomized trials and thousands of updated systematic Cochrane reviews of trials [1].

There are several reasons why doctors are unable to choose the best treatments for their patients and the biggest obstacle for evidence-based healthcare with prudent use of resources is that research results are being reported selectively.

Another important problem is that the drug industry is not obliged by law to compare its new drugs with the best existing drugs but can obtain marketing approval by comparing with placebo. It even suffices to demonstrate a statistically significant effect in two placebo-controlled trials, even though the drug might not have worked in many other placebo-controlled trials. Financial success would be difficult if a well-conducted trial showed that a new expensive drug is not any better than an old cheap one, or is worse. Head-to-head comparisons of drugs can be subject to bias, in all phases of a trial, in design, analysis and reporting [2-8], and can cause the reported results to be misleading. Data sharing cannot resolve all problems, but it would make it possible to demonstrate many of the hidden flaws in the research process.

## Selective reporting

Comparisons of published drug trials with unpublished trials or other data available at drug regulatory agencies have shown that the benefits of a number of drugs have been much overrated [6,9,10] and the harms much underrated [11]. This is a universal problem that has been documented across many different drug classes [10]. The effect of antidepressants, for example, was 32% larger in the published trials than in all trials submitted to the US Food and Drug Administration (FDA) [9].

Another review of antidepressants showed that the statistical analyses in published reports were considerably more favorable for the drugs than those required by law to be submitted to the drug regulatory agency [6]. The published analyses were mainly 'per protocol analyses,' where patients who drop out of the trials, for example because of lack of effect or adverse effects, are not accounted for. Those required by law are 'intention to treat analyses,' which are far more reliable, as they include these patients.

The effect of antidepressants is relatively small [12] and they have important adverse effects that, however, to a large extent have been hidden. Adverse events of suicidal thoughts and behavior, and even suicide attempts, in patients taking antidepressant drugs have gone unreported by the events being coded as emotional lability, admissions to hospital, treatment failures, noncompliant patients, or drop-outs [11,13]. Furthermore, several companies were reported to have added suicidal events to the placebo group, although they had not occurred while the patients were on placebo [11,14]. Only full access to trial data can reveal these problems.

When the UK National Institute for Health and Clinical Excellence (NICE) was drafting guidelines for childhood depression they observed that, based on the published trials, they would recommend antidepressants, but based on all the trials, including the unpublished ones, they would not [15]. NICE does not have a legal right to access the unpublished reports at the UK Medicines and Healthcare products Regulatory Agency (MHRA), but in this case, the Agency made the reports publicly available.

Similar examples abound in all therapeutic areas and studies have shown that even for those trials that do get published, trial outcomes are often reported selectively, or the data are massaged and changed until they say something positive [2-11,14,16-21]. Some of this was revealed in US court cases [20,21], and some because scientific-ethical committees or public funders made trial protocols available for researchers wanting to compare them with published reports [16-18].

Although it seems to be less common, selective reporting is also widespread in clinical trials performed by academics independently of the drug industry [16,17]. When the results of three published trials comparing two techniques for hernia surgery were combined, one technique was significantly better than the other, but when the trialists shared their unpublished data, there were 13 trials and the second technique was now significantly better than the first [22].

## Consequences of selective reporting

Selective reporting can have disastrous consequences. For example, class 1 anti-arrhythmic drugs likely caused the premature death of about 50,000 Americans each year in the 1980s [23]. An early trial found nine deaths on the drug and only one on placebo, but it was never published [24].

More recently, the anti-arthritis drug, rofecoxib (Vioxx), a COX-2 inhibitor, likely caused about 100,000 unnecessary heart attacks in the US alone [25,26], corresponding to about 10,000 deaths, which could largely have been avoided by using other, equally effective drugs causing less harm. Several drug companies misrepresented the risk of harm with their COX-2 inhibitors. Cases of myocardial infarction and deaths with rofecoxib, for example, were missing in reports of the pivotal trials [27-30] and Merck selectively targeted doctors who raised questions about rofecoxib and pressured some of them through deans and department chairs, often with the hint of loss of funding [31].

Pfizer denied that celecoxib causes heart attacks at an FDA hearing in 2005 [32], despite having unpublished evidence to the contrary [32] and still said the evidence was not conclusive in 2009 in information to patients invited to take part in a trial [33].

Misleading 6-month data in two pivotal trials of celecoxib, funded by Pharmacia, were published [34,35]. The publications showed that celecoxib induced fewer gastrointestinal ulcers than its competitors, but it was later revealed that the trials ran for longer than 6 months, and analyses done according to the trial protocol showed no advantage of celecoxib [34]. Despite the fact that only 16 out of at least 27 trials of celecoxib were included in the relevant FDA reports [36], independent researchers who had access to FDA data nevertheless substantiated the cardiovascular harms of celecoxib [37].

## Non-transparent data on lethal harms

A drug company applying for marketing authorization for a new drug often submits tens of thousands of pages describing the results of its clinical trials, the clinical study reports. Drug regulatory agencies do not have the capacity to scrutinize so much material and therefore need to trust the companies' handling of the data and the summaries and tables in their reports.

However, several drug companies have been reported to have hidden studies and results showing that their drugs cause lethal harms [38-43].

The US Freedom of Information Act has played an important role in reducing the exposure of the population to potentially harmful drugs. In 2005, an FDA advisory committee recommended approval of an antidiabetes drug, muraglitazar, but independent researchers who analyzed the submitted trial data found that the original analyses supporting the FDA application were flawed, and that the drug was harmful [44,45].

Rosiglitazone is another example. Antidiabetes drugs are supposed to lower cardiovascular mortality but rosiglitazone increases it. This was known by GlaxoSmithKline for many years but the company failed to warn the regulatory authorities and the public. Instead, it intimidated physicians raising uncomfortable questions about the drug [46].

In 1999, the company, then known as SmithKlineBeecham, completed a trial that found more cardiac problems with rosiglitazone than with pioglitazone [47]. 'These data should not see the light of day to anyone outside of GSK,' according to an email from a SmithKline executive, that was reported in the New York Times [47].

In 2004, the WHO sent GlaxoSmithKline an alert about cardiac events and the company performed a meta-analysis that confirmed this, which it sent to the FDA and the European Medicines Agency (EMA) in 2006, but the drug regulatory agencies did not make the findings public because of the proprietary nature of companies' trial results [48].

Also in 2004, the Attorney General of New York State accused GlaxoSmithKline for 'repeated and persistent fraud' in relation to concealing harms of its antidepressant drug, paroxetine [49]. A subsequent legal settlement required the company to post results of its clinical trials [50]. This enabled independent researchers to have a closer look at rosiglitazone, and in a 2007 meta-analysis of 42 trials, 27 of which were unpublished, they showed that the drug causes myocardial infarction and cardiovascular death [51].

When these researchers submitted their paper for publication, an academic peer reviewer broke the rules and faxed the manuscript to GlaxoSmithKline [52]. The company unblinded its ongoing RECORD trial, that the EMA had required the company to carry out because of cardiovascular safety concerns [52]. However, Home *et al*., funded by GlaxoSmithKline, published a preliminary analysis electronically in the *New England Journal of Medicine *only two weeks after the independent researchers published their meta-analysis in the same journal. Home *et al*.regarded their findings as 'inconclusive' [53], and when final results were published in *The Lancet *two years later [54], they appeared to be false [52]. Since the 1950s, FDA rules have required drug companies to turn over all individual patient case reports from their clinical studies. This permits re-analysis of how each case was coded [48], and it enabled Marciniak, an FDA scientist, to scrutinize the RECORD trial data [55]. The EMA had accepted the company's findings that the risk of complications was the same, 14.5% for rosiglitazone and 14.4% for the comparator [53]. However, Marciniak studied 549 case reports and found many missing cases of cardiac problems that favored rosiglitazone four to one and an increased risk of myocardial infarction [55]. For one patient, there were 1,438 pages, and for most of the other 4,500 patients there were several hundred pages, making a review of all case reports a huge task [48]. Marciniak concluded that the case report forms are essential for understanding a study and found that also in the RECORD trial, rosiglitazone increased cardiovascular risk [48,55].

Rosiglitazone was suspended in Europe in September 2009 whereas it remained on the market in the US. In an unprecedented move, the FDA invited additional people to its 2010 advisory committee meeting - to decide if the drug should remain on the market - who had been involved in the 2007 meeting but were no longer active members of either committee and some of whom had previously voted in favor of the drug remaining on sale [56].

## Oseltamivir (Tamiflu) against influenza

Oseltamivir (Tamiflu) against influenza illustrates that lack of access to full clinical trial data can be extremely wasteful. During the rather mild influenza epidemic in 2009, European governments stock piled Tamiflu. Tamiflu and similar drugs were promoted by the WHO as a key part of influenza prevention and treatment, but most of the drug supplies were never used [57].

The belief in the efficacy of Tamiflu hinged on trials that were only published as a company-sponsored meta-analysis. When independent researchers succeeded to get access to some of the data it turned out that the effect of the drug in preventing serious complications, above all bacterial pneumonia, was unclear, and that billions of Euros had been wasted [58]. These researchers concluded that we should 'regard any industry sponsored trial published in journals as marketing, unless proved otherwise' [58]. Indeed it was revealed that the scientific papers on Tamiflu were not written by the academics listed in the byline but by medical writers that liaised directly with Roche's marketing department (although at least one of the authors denied that their paper was ghost-written) and who were told that they had to come to the conclusion that Tamiflu was the answer to the influenza epidemic [57].

It was also remarkable that the EMA stated in its summary of product characteristics that Tamiflu reduced influenza complications significantly (*P *= 0.001), whereas the FDA gave the opposite view when it stated on the product label that Tamiflu had not been shown to prevent complications [57]. If Tamiflu does not reduce complications, there is little rationale for using this expensive drug.

## International calls for data sharing

Sharing knowledge has led to tremendous benefits for our societies. One need only think of Google and Wikipedia, which contains more than 3.5 million articles and would amount to 1,400 volumes if printed in the same format as the *Encyclopaedia Brittanica *[59].

Few things matter more to people than their health. It is therefore counterintuitive that researchers working with patients are generally very secretive about their data. In genetics, molecular biology, the social sciences and in many other areas of the life sciences, researchers routinely share their data [60].

A public that pays for most medical research through taxes and public funds is becoming increasingly puzzled by the barriers that deny access to the results of that research [61]. This had led to calls for transparency and access to the research protocols, the results and the raw data from major international organizations, politicians, funders, editors of medical journals and researchers, particularly during the last decade.

Appendix 1 describes such calls from the ministerial meetings in Mexico City and Bamako, the World Bank, the WHO, the OECD, the European Commission, the UK National Institute for Health Research, the Wellcome Trust, the Bill and Melinda Gates Foundation, the Hewlett Foundation, the US Congress, the US National Institutes of Health (NIH), the US National Science Foundation, the US Centers for Disease Control and Prevention (CDC), *Science*, *Nature *journals, *The Lancet*, *British Medical Journal (BMJ)*, *Annals of Internal Medicine*, *The Public Library of Science (PLoS) *Journals, and The Cochrane Collaboration [62-87].

The European Commission favors open access publishing, where the authors pay for publication while access is free for the users. It observed that journal subscription prices increased substantially above inflation level, which is particularly acute for less well-endowed institutions and in countries with lower income levels. In addition, several big publishers cut access to thousands of journals in poor countries provided through the WHO-initiated HINARI (Health InterNetwork Access to Research Initiative scheme, worsening considerably the global inequities in health and healthcare research [88].

## Drug regulatory agencies

Drug regulatory agencies have been the notable exception to these calls for data sharing. They have generally not been willing to share data of pivotal importance for public health and rational decision making with the citizens. Before 2010, it was impossible for researchers to get access to unpublished trial reports and their corresponding protocols from the EMA, although, according to Regulation (EC) No 1049/2001, a basic principle in the European Union is to allow its citizens the widest possible access to the documents its agencies possess [89,90]. This openness 'enables citizens to participate more closely in the decision-making process and guarantees that the administration enjoys greater legitimacy and is more effective and more accountable to the citizen in a democratic system.' It also 'contributes to strengthening the principles of democracy and respect for fundamental rights' [89,90].

In 2010, a colleague and I from The Nordic Cochrane Centre were granted access to trial protocols and clinical study reports for two anti-obesity drugs at the EMA but it took us three years and a complaint to the European ombudsman to get there. The EMA put forward several arguments to avoid disclosing the documents: protection of commercial interests, no over-riding public interest, the administrative burden involved, or the worthlessness of the data to us after the EMA had redacted them [90].

The ombudsman inspected the files at the EMA and concluded that the documents did not contain commercially confidential information. After he accused the EMA of maladministration in a press release, three years after our request, the EMA reversed its stance. The EMA now gave the impression that it had favored disclosure all the time and agreed with the ombudsman's reasoning.

The ombudsman did not take a definitive position regarding whether the presence of 'personal data' could entitle EMA to redact the documents. He noted that they do not identify patients by name but by their identification and test center numbers, and he concluded that the only 'personal data' are those identifying the study authors and principal investigators.

This is very important progress for patient safety. Researchers can now get access to the detailed lists of individual patient data in clinical study reports, enabling them to perform an independent interpretation of the harm of drugs.

Our case has set an important precedent, and we recommend that FDA and other drug regulatory agencies follow suit [90]. Only a few years ago, researchers found that many pages for a COX-2 inhibitor, valdecoxib, had been deleted from FDA documents, when they were allowed access, because they contained 'trade secrets and/or confidential information that is not disclosable' [36]. Suicides, occurring while patients take a drug with the aim of preventing suicides, should not be regarded as a trade secret [91]. Furthermore, indirect patient identifiers such as body weight, blood pressure, asthma severity or descriptions of harm, are not, in isolation, 'personal data.'

I am convinced that virtually all trial participants would be interested in letting others have access to their anomymized data, given that this enables us to get a more truthful view of the benefits and harm of drugs and other interventions. Furthermore, despite theoretical concerns about the possibility of identifying individuals in shared data sets, no breaches of confidentiality have yet been recorded in anonymized data sets, as far as I am aware.

Data sharing will lead to greater transparency in drug regulatory processes, which is much needed, and should reduce potentially lethal consequences for the patients.

Data access adds considerably to the essential role drug regulatory agencies have as a guardian of public health, as many citizens have the interest, skills and time to scrutinize the clinical documentation to a degree and detail drug regulatory agencies cannot do. In fact, they are under political pressure to work faster for the benefit of the companies and the national economies. This focus on regulatory speed rather than on quality and patient safety is relatively recent [92,93], but it has already had lethal consequences [94]. Of drugs approved in 1997 to 2000, 5.3% were later withdrawn from the market because of serious harm, compared with 1.6% of drugs approved in 1993 to 1996 [92,94]. Drugs approved just before the official FDA review deadline were more likely to be withdrawn from the market than drugs approved after the deadline [95,96].

In 2009, the FDA admitted that its former commissioner had unduly influenced the decision process for approving a patch for knee injuries [97]. FDA scientists had repeatedly and unanimously over many years decided that a particular medical device was unsafe, but after extreme, unusual and persistent pressure from four congressmen, FDA managers overruled their own scientists and approved the device. These congressmen had received campaign contributions from the company marketing the device [97].

When an associate director in the FDA's Office of Drug Safety, David Graham, showed that rofecoxib increases the risk of serious coronary heart disease, his study was pulled at the last minute from *The Lancet *after one of his directors had raised unfounded allegations of scientific misconduct with the editor [98,99]. The study was later published [100], but just a week before Merck withdrew rofecoxib from the market, senior people at the FDA questioned why Graham studied the harm of the drug, because the FDA had no regulatory problems with it, and they also wanted him to stop doing this [100]. Graham needed congressional protection to avoid getting fired from the FDA [100]. Other safety officers at the FDA have also been prevented from presenting their findings of the lethal harms of drugs [39,93,98]. A 2006 survey showed that 70% of FDA scientists are not confident that products approved by the FDA are safe [101].

## Research ethics

It is important to note that parts of the Nuremberg code and the Declaration of Helsinki are not only regarded as universal codes of ethics, but are to be considered a 'customary international law norm' [102].

Selective reporting violates the Declaration of Helsinki [103], which says that, 'Authors have a duty to make publicly available the results of their research on human subjects.' Failing to do so is unethical for a number of reasons.

When less favorable results and adverse effects are not published, the standard of care inevitably suffers. Further, informed consent forms do not tell trial participants that the sponsor may decide not to publish. In fact, they are usually told that the data they provide will add to existing knowledge, which is misleading because the chance of publication depends on the magnitude and direction of the results [16-19]. Thus, patients are being exploited for commercial or career gains, that is used as a means to an end.

The Declaration says that, 'Medical research involving human subjects must ... be based on a thorough knowledge of the scientific literature.' If the knowledge base is incomplete, some patients will suffer and die unnecessarily. Researchers may for example include patients in trials of similar compounds as one that has been shown to be deadly because they are unaware of this [24]. An incomplete knowledge base also leads to redundant research, which by its very nature is unethical, and informed consent is an illusion when patients and their doctors can only get access to biased information. New research should not be done unless, at the time it is initiated, the questions it proposes to address cannot be answered satisfactorily with existing evidence [104].

Clinical research relies on the patients' altruistic willingness to contribute to advancing science for the public good [105], but research can only be a public good, if the public can see the data. It is curious that trial participants are willing to share data about themselves with the investigators when the investigators are unwilling to share these data with trial participants and others but regard them as their personal property. This double standard expresses a lack of respect for the trial participants, which is particularly pronounced in industry-sponsored trials. A review of trial protocols showed that the sponsor either owned the data, needed to approve the manuscript, or both, in half the cases, and none of these constraints were stated in any of the trial publications [106]. Even researchers who have contracts permitting them to publish, or who do not collaborate with the drug industry, may face legal threats if they wish to publish papers that are not in the industry's interest [107].

Such deliberations make it clear that it is a moral imperative to render all results from all trials involving humans, including healthy volunteers, publicly available. This should apply to all research, for example also to pharmacokinetic studies, as these could suggest that a drug would be hazardous to use in the elderly or in certain patient groups because of reduced drug metabolism.

Respect for trial participants who often run a personal and unknown risk by participating requires that they, and therefore also the society at large that they represent, be seen as the ultimate owners of trial data. We should no longer accept that data generated by patients are treated as the private property of investigators or drug companies, which aim to maximize their publication record or income at the expense of the widest possible use of the data [60]. It is also unacceptable that in virtually all industry-sponsored trials, the only people who have seen the entire data set are company employees.

Also, the trial protocols need to be publicly available. Patients wishing to see the protocol or get a copy of it when asked to participate in a trial usually cannot get it, as the drug industry regards their protocols as confidential. This confidentiality clause is unnecessary, as there is nothing in a trial protocol that with any reasonable justification can be regarded as commercially sensitive information [16,90], for example there is nothing about how a drug is manufactured.

## Benefits of data sharing

Data sharing would lead to tremendous benefits. First, we would become much better informed about what the true benefits and harms of our interventions are, which would lead to better results with less harms throughout healthcare.

Second, the incentive for cheating would be much reduced, as it would be a risky affair when others can check the methods and calculations against the trial protocol and the raw data.

Third, the efficiency of healthcare research would be much improved, as many important research questions can be answered by using existing data, sparing researchers and patients from collecting new data, and saving funds for better purposes.

Fourth, access to raw data would make meta-analyses of trials studying similar interventions and patient groups much more reliable than if based on published summary data. It would also allow exploratory analyses aimed at identifying subgroups of patients where the treatment would be particularly beneficial or where it might be harmful, resulting in much more cost-effective and evidence-based use of interventions, with large savings for our societies.

## Harms of data sharing

The most obvious harm of data sharing is that anyone with an agenda could selectively interpret the data in a way that furthers this agenda, for example plaintiffs' lawyers and anti-vaccination proponents. But consider the alternative. Societies that have only one official version of the truth are not societies we would like to live in. Equally important, it is difficult to imagine a worse situation than the status quo, where people with vested interests distort the evidence for commercial or career gains so often that it seems to be the norm, rather than the exception [2-11,16-20]. By allowing free access to the data, this situation could only improve, as all those who wish to get closer to the truth than is currently possible would be able to correct the published or institutionalized record.

## Commercial interests

The arguments in favor of data sharing are so strong that it would seem difficult to argue convincingly that we should not share our data. There is something fundamentally wrong with our ethics and priorities in healthcare if commercial success is dependent on withholding data that are needed for rational decision-making for patients, doctors, other health professionals, and the politicians guarding the public purse to which we all contribute.

Commercial interests seem to be incongruous with data sharing. Publication rights on industry-initiated trials have been constrained, which is inappropriate in clinical research [106]. A scientist involved with European drug regulation concluded that the only reasonable explanation for the confidentiality arguments was that the 'industry wants to avoid any discussion about the amount and quality of the data they provide to justify marketing of drugs. There is also the complicity of the regulatory agencies that have access to the data, but avoid making it public, possibly so as not to be questioned over their decisions?' [108].

Access problems to industry data and publication bias were highlighted when the German Institute for Quality and Efficiency in Health Care (IQWiG) recently asked Pfizer to submit a list of all trials performed with its antidepressant drug, reboxetine. Pfizer delivered an incomplete list of trials, which from Pfizer's point of view were 'suited for a benefit assessment' [74]. When independent researchers were eventually able to analyze both published and unpublished data for a meta-analysis, reboxetine, which is marketed in several European Union (EU) countries, was found to be ineffective or potentially harmful [109].

It has been argued that it is competition between commercial companies that drives the discovery of new treatments, but many major breakthroughs in healthcare have come from publicly sponsored research [110,111]. The drug industry has been reported to spend more on marketing than on research [110]. Moreover, drugs have been sold at prices our societies can hardly afford, and new drugs rarely offer substantial advantages over existing drugs and may cost several times, in one case 350 times, as much [112].

Arguments about competitiveness are unconvincing for other reasons. As noted by The British House of Commons Health Committee, society's obligations towards the patients who participate in trials, and all other patients, must take precedence over commercial interests [113]. And the competitiveness argument can be used to support almost any doubtful practice, including allowing selective publication of data and trials. Further, I am aware of no evidence to suggest that increased openness and transparency would negatively impact drug development and profitability.

As a general principle, data sharing would not be anti-competitive, as all companies would be affected equally by it. It would only lead to competition at a higher ethical level, with the added benefits of more transparency, less potential for fraud, more rational use of resources, and less potential for harm.

It is also of note that drug development is deeply dependent on, and a direct consequence of, patients' willingness to volunteer for clinical trials. Through these volunteers it might be discovered that a drug is too hazardous to market, or the right marketing niche for a product may be defined [11].

This means that it is artificial to distinguish between industry-sponsored trials and publicly-sponsored trials, although most calls for data sharing have only alluded to the latter. The public is always a partner, contributing not only trial participants, but also the infrastructure needed for the research. Further, taxpayers contribute substantially, both to research and by reimbursing drugs once they are on the market.

The same rules ought to apply to industry-sponsored trials as to publicly-sponsored trials, which would help re-instate public trust in industry-sponsored research, which is at a historically low level at present [113]. This can only occur if the industry discloses all protocols, results and raw data from all studies in humans, including those in non-patient volunteers who may also suffer the harm of the drugs [11,114]. In a 2006 phase 1 trial, six healthy volunteers treated with a very small dose of a monoclonal antibody that had not caused problems in animals developed catastrophic systemic organ failure. The investigators succeeded to rescue them all despite their serious condition, but it required cardiopulmonary support at an intensive care unit [114].

The current lack of access to data also has untoward consequences for the drug industry, as it leads to a huge waste of resources. When failures with previous drugs are kept secret, expensive development programs for similar drugs can go on for years after they would have been stopped if the data had been known. Openness therefore has potential benefits for drug innovation.

Finally, it can be argued that if companies or academic researchers are not willing to share their raw trial data, it may raise a suspicion of improper research methods. Their publications should therefore not be seen as science but as advertisements, that is as vehicles for financial gains or career advancement. We should strive towards a future in which such trials, where raw data are not available, are routinely excluded from systematic reviews, meta-analyses and evidence-based guidelines. At present, we may abstain from concluding anything about the possible merits of the intervention, when data are being withheld, as researchers recently did with respect to oseltamivir and reboxetine (see above) [57,74,109].

## Academic interests

The primary concern of doctors and other health professionals must be their patients. I cannot envision any reasonable argument against data sharing that overrides the benefits for the patients of data sharing. It is not even a problem for the academics' careers, in fact quite the contrary. International calls for data sharing have noted that data sharing should be valued when judging the performance of academics, and leading journal editors, members of the International Committee of Medical Journal Editors, have agreed that posting trial results in tabular forms in the same register where the investigators registered their trial will not prevent them from publishing the results in journals [115].

Academics must ensure that they have complete access to all data, which is very rarely the case for industry-sponsored trials despite claims to the contrary in published papers, for there to be genuine research partnerships, and they should not put their names to publications if this is not the case.

## Necessary legislation

It has been amply documented that guidelines and other voluntary agreements about data sharing do not work. For example, although the PLoS journals require of authors that, 'data should be made freely available upon reasonable request,' researchers who asked ten authors for their data, not to challenge their original conclusions, but to test a new hypothesis, only obtained one original data set [116].

If national or supranational legislation impedes the much-needed reforms, the laws should be changed, harmonizing national laws. The EU legislated in 2009 that 'protocol-related information' and all results from trials in children must be made publicly available no more than six months after the trial ended and must be submitted electronically [70]. It would be unreasonable to defend a position that only details on trials in children and not those on trials in adults must be made publicly available.

Data sharing should mean that the data can be used for whatever purpose other researchers might find relevant, without needing to obtain permission from those who assembled the data. This is in agreement with the views expressed by the OECD, the European Commission and the US CDC [66-68,70,79]. Further, there should be no restrictions, for example no requirement of co-authorship as a condition for receiving data [78], apart from those that may be needed to prevent identification of particular patients, which is rarely a problem, as patients are listed by their trial numbers only.

Data access must be free of charge. When Statistics Canada attempted to recover costs for its data management by charging data users, the use of these public data decreased dramatically [67]. User fees would impede research particularly much in low-income countries.

Registration of clinical trials is already a legal requirement in some countries including the US and Brazil [62]. All clinical trials involving humans must be registered at a public register, for example the WHO register, http://www.who.int/trialsearch[62], or the US http://www.clinicaltrials.gov, before the first trial participant is recruited. Registration should include:

1) The full clinical trial protocol and any subsequent amendments, with dates. This will allow other researchers to assess whether manipulations with the data have occurred during data analysis and report writing. Such manipulations, and many other important discrepancies between protocols and published reports, are very common [117]. A cohort study of 102 clinical trials, many of them multinational drug trials, showed that at least one primary outcome was changed in two-thirds of the cases between the protocol and the publication, and this was not mentioned in any of the publications [16]. It is deceptive to make changes in primary outcomes without telling the readers about it, as the target is moved after the results are known. In line with the European ombudsman's observations [90], we did not find any information that could compromise commercial interests in trial protocols of industry-initiated trials [16].

2) The clinical record forms. These are very important, as the obtained results depend on the way questions are being asked and recorded [11,118].

3) Any separate agreements between sponsor and investigators that are not included in the protocol, for example on finances, ownership of data and publication rights. Thirteen of 44 protocols of industry-initiated trials from 2004 referred to a separate publication agreement, compared with none of 44 protocols from 1994-1995, and none of these agreements were submitted to the Scientific-Ethical Committees [106].

Clauses in trial protocols that give the sponsor ownership of the data, or state that manuscripts need to be approved by the sponsor, should be made illegal. As contract law trumps every other form of law, informed consent forms should be written as a contract that states explicitly that the public owns the collected data; that all data will be made available to the public in an anonymized fashion; and that the data can be used for other purposes than for meeting the trial's objectives. This should not be a problem, as it has always been an implicit understanding when patients volunteer for trials that the data may be used for additional purposes, and many of these spin-off projects were not even thought about when the trial was planned.

Similarly, data sharing for old trials do not violate the patients' rights or interests. It should therefore not be required that patients be asked whether they accept that old data be shared with other researchers, which would make a lot of potentially fruitful research impracticable.

It should be a legal requirement to provide all results and raw data within an appropriate period of time, which, in accordance with most calls for data sharing, should be no later than 12 months after the randomized phase of the trial ended [69,70,73-75]. These data should be made available in publicly administered data repositories, preferably the trial register (if not, there must be a link from the register to the repository). If needed for using the data, they must be accompanied by the statistical codes. Raw data about adverse events should mean the original descriptions, exactly as reported narratively by patients or researchers on the case report forms, before any coding or adjudication has taken place for categorization purposes.

Drug regulatory agencies should be required to make available the protocol, the clinical study report, separate agreements, and the raw data on their website. With rare exceptions, a drug should not be launched until these data have been available for 12 months, enabling doctors, patients and those who consider whether to reimburse the drug with taxpayers' money to have their own look at the data [119].

Research ethics committees should require registration of trials as a precondition for ethics approval, and should ensure that all trial results, protocols, separate agreements, and raw data are publicly disseminated and registered with the committees that approved the trial no later than 12 months after the trial ended.

Research ethics committees should also require a systematic review of similar previous trials in the application, as the UK National Institute for Health Research does [120], and as the Danish ethics committees required in 1997 [121,122]. A summary of relevant systematic reviews should be made available to trial participants [123]. It is impossible to know whether a proposed trial is redundant without such a review, which might need to be updated while the trial is running, as new evidence could render continuation of the trial unethical.

There are numerous examples of unethical research where one group received placebo, although a systematic review would have shown that the studied treatment was life saving, for example antibiotic prophylaxis for colon surgery, thrombolysis for myocardial infarction and aprotinin for perioperative bleeding [123-126]. Previous, relevant trials are often omitted from published reports of trials. For example, in the published reports of placebo-controlled trials with aprotinin, only 20% of previous trials were cited, and only 15% of the reports cited the largest trial, which was 28 times larger than the median trial size [126]. A study from 2011 that included 227 meta-analyses of 1,523 trials published between 1963 and 2004 showed that fewer than 25% of the previous trials had been cited [127]. This suggests that most previous trials have also been omitted from the trial protocols because if they had been cited, there would be no reason not to cite them also in the trial report.

The data must be made accessible in electronic formats wherever they are stored. Access must be easy and it should be easy to work with the documents. Access only to tens of thousands of pages of paper is an unacceptable obstacle to the progress of clinical research.

## Sanctions

The British House of Commons Health Committee examined the drug industry in detail in 2004 to 2005 and found that its influence was enormous and out of control [113,119]. However, although the Members of Parliament felt that the UK Medicines Agency was not competent to undertake its duties as a guardian of public health, the Government declined a public hearing.

Many violations of the law in the context of clinical research or marketing have been documented [11,38-41,92,107,110,111,128-137]. A 2010 study showed that the drug industry was the biggest defrauder of the US Government under the False Claims Act and that the civil and criminal settlements had increased dramatically in the past five years [138].

Pfizer agreed in 2009 to pay US$2.3 billion to settle charges of fraud and civil and criminal liability over its promotion of off-label use of four drugs [139]. The US Department of Justice said it was the largest healthcare fraud settlement in the Department's history and the largest criminal fine ever. Even so, this was reported to be equivalent to less than three weeks of Pfizer sales. As part of the settlement, Pfizer entered a corporate integrity agreement with the Office of the Inspector General of the Department of Health and Human Services to avoid and detect such problems. Pfizer previously entered three such agreements [139].

Violations of a new law about data sharing should therefore result in more tangible measures if they are to have any effect. I suggest that those who violate the law, whether it be companies, research groups or individual researchers, receive a fine corresponding to 10% of last year's gross income before taxes, and will be prohibited from doing clinical research in the jurisdiction involved, for example in all EU countries if the violation occurred in one of them, for an appropriate period of time, for example two years for first-time offenders.

In serious cases, or if repeated violations occur, or if the lack of compliance with the law is not remedied within a certain period of time, the sanctions should be more severe, including the possibility of suspending the marketing authorization, if the violation concerns a marketed drug or medical device.

Any violation of the law should also mean that new projects from the same sponsors or researchers cannot be approved by the research ethics committees until all data from previous studies have been made public.

## Conclusion

Data sharing in clinical research is recommended by many of the world's leading scientific institutions and policy makers, most recently also by The Cochrane Collaboration [140]. It is a moral imperative and we should act now, as it will empower citizens and convey tremendous scientific, economic, and social benefits [67]. Guidelines exist on how this can be done without compromising patient anonymity [141].

## Appendix 1. Recent international calls for data sharing

In 2004, The Ministerial Summit on Health Research in Mexico City stated that, 'Research results must be published, documented in internationally accessible registers and archives, and synthesized through systematic reviews. These actions can help to inform decisions about support for new research and to build public confidence in science' [62]. The Summit called for action by 'All major stakeholders, facilitated by the WHO Secretariat, to establish a platform linking a network of international clinical trials registers to ensure a single point of access and the unambiguous identification of trials.'

In 2008, the Global Ministerial Forum on Research For Health in Bamako agreed 'To develop, set, and enforce standards, regulations, and best practices for fair, accountable, and transparent research processes' and 'the registration and results reporting of clinical trials, and open and equitable access to research data, tools, and information' [62,63].

In 2010, the World Bank announced that it would make its data base open access, and would adopt disclosure as the default position for both data and documents relating to lending [64].

In 2010, the WHO noted that, 'public accountability of research is not keeping pace with best practices,' and lamented that, 'The opportunity of creating a shared framework for storing and sharing research data, tools and materials has not been seized with the same energy in the area of health as it has in other scientific fields, and policy-makers are neither contributing to research priorities nor using evidence to inform their decisions' [65]. The WHO recommended sharing research data and having open access to the results, partly because failure to obtain it can be literally fatal [61].

The OECD declared in a 2004 report that, 'Co-ordinated efforts at national and international levels are needed to broaden access to data from publicly funded research,' recognizing that, 'open access to, and unrestricted use of, data promotes scientific progress and facilitates the training of researchers,' and that, 'open access will maximise the value derived from public investments in data collection efforts' by being 'put to use for multiple research purposes by many research institutes of the global science system, thereby substantially increasing the scope and scale of research,' seeking 'transparency in regulations and policies,' and 'reducing unnecessary barriers to the international exchange of these data' [66]. These principles were set into perspective in a journal article [67].

The European Commission refers to the OECD declaration [66], stating in 2007 that, 'Fully publicly funded research data should in principle be accessible to all' [68]. It aims to 'maximise the socio-economic benefits of research and development for the public good' and recognizes the need for access not only to journal articles but also to research data, which would help accelerate innovation and avoid duplication of research efforts. The Commission furthermore notes that, 'The public purse pays for research, peer review (through reviewers' salaries), and journals (for example through library budgets). It is natural that public actors should request a better return on their investment.' It is envisaged, but not yet implemented, that the EudraCT Clinical Trials Database - where all EU drug trials must be registered - should also contain the results, within 12 months after the termination of the trial, in accordance with article 57(2) of regulation (EC) No 726/2004 and its implementing guideline 2008/C168/02 [70].

The UK's Medical Research Council policy [71] builds on the OECD principles [66], acknowledging that publicly-funded research data are a public good, produced in the public interest, which should be openly available to the maximum extent possible.

Similarly, the Wellcome Trust believes that, 'success in maximizing the value of research data depends crucially on fostering a culture in which both data generators and data users adopt good research practice, and act with integrity and transparency in managing, using and sharing research data' [72]. It requires a data management and sharing plan to be built into grant proposals when 'the proposed research is likely to generate data outputs that will hold significant value as a resource for the wider research community. '

In 2007, the US Congress directed the NIH to require sponsors to post the 'basic' results data from clinical trials (other than phase 1 trials) on drugs that have been approved by the FDA. The FDA required in 2007 that the results of certain clinical trials be posted on the internet within a year of the trial's completion, even if the results had not yet been published in a scientific journal [73,74]. The law does not provide access to the full study protocols and it does not cover trials completed before 27 September 2007 or trials of drugs that were never approved [74].

In 2008, the US Congress approved the Consolidated Appropriations Act, which gives the public access to the published results of NIH-funded research. The policy requires that these papers are accessible on the open, publicly-owned resource, PubMed Central, no later than 12 months after publication [75].

The US NIH has declared that, 'Data sharing is essential for expedited translation of research results into knowledge, products and procedures to improve human health' [77]. In 2003, the NIH decided that all investigators seeking more than $500,000 in grant support per year should make available their 'final research data, especially unique data ... to other investigators.' Furthermore, 'Data sharing should be timely and no later than the acceptance for publication of the main findings from the final dataset.' Most importantly, NIH declared that, 'When making data available, researchers cannot place limits on questions or methods nor require coauthorship as a condition for receiving data'[77]. About protecting the rights and privacy of human subjects, NIH wrote that, 'data sharing is possible without compromising these efforts because identifiers can be removed from data' [77].

The US National Science Foundation has a data archiving policy that is very similar to that of the NIH being 'committed to the principle that the various forms of data collected with public funds belong in the public domain,' and noting that, 'The purpose of this policy is to advance science by encouraging data sharing among researchers. Data sharing strengthens our collective capacity to meet scientific standards of openness by providing opportunities for further analysis, replication, verification and refinement of research findings' [78].

The US Centers for Disease Control and Prevention (CDC) 'believes that public health and scientific advancement are best served when data are released to, or shared with, other public health agencies, academic researchers, and appropriate private researchers in an open, timely, and appropriate way. The interests of the public - which include timely releases of data for further analysis - transcends whatever claim scientists may believe they have to ownership of data acquired or generated using federal funds. Such data are, in fact, owned by the federal government and thus belong to the citizens of the United States' [79]. The CDC states what the advantages are of data sharing [79]:

- improve the quality of CDC data and the consistency of data across CDC,

- ensure that CDC scientists, contractors, awardees, and grantees are held accountable for their findings,

- provide opportunities for study results to be validated,

- uncover new areas for research,

- improve public health practitioners' understanding of various research methods,

- encourage analysts from other disciplines (for example, economists, social scientists) to examine public health questions, and

- build trust with outside partners and the public by allowing an open critique of CDC investigations.

It also notes that, as a public health agency, CDC is accountable to the public for the data it produces through research, and CDC scientists are accountable for their work, and their findings are subject to independent validation. The principle is to release the data for public use without restrictions within a year after the data have been evaluated for quality and shared with any partners in data collection.

In 2010, a number of funders, among them the WHO, the World Bank, the US NIH, the US CDC, the Bill and Melinda Gates Foundation and the Hewlett Foundation, agreed on data sharing, as it would generate faster progress in improving health, better value for money and higher quality science [80].

*Science *notes that, 'appropriate data sets ... must be deposited in an approved database' [81]. *Nature *journals state that, 'authors are required to make materials, data and associated protocols promptly available to others without preconditions. Data sets must be made freely available to readers from the date of publication, and must be provided to editors and peer-reviewers at submission, for the purposes of evaluating the manuscript' [82].

The *Lancet *requires submission of the trial protocol in addition to the trial report [83]; and the *British Medical Journal (BMJ) *asks authors to include a data sharing statement at the end of each original research article, arguing that many people would call this a moral obligation because most research is publicly funded and involves the public as participants [84].

*Annals of Internal Medicine *also has a data sharing statement, and trials have been published for which the study protocol, the raw data, and the statistical code needed for analyzing the data are available on a website [85].

The *Public Library of Science (PLoS) *journals, which are open access journals, require that the underlying data be made immediately available without restrictions and encourage researchers to contact the editors if they encounter difficulties in obtaining the data, in which case the editors may post corrections on articles, contact authors' institutions and funders, and in extreme cases withdraw publication [86].

In 2004, The Cochrane Collaboration recommended legislation to mandate prospective registration of trials in a register as a condition of funding, ethics and regulatory approval to ensure that all trial results become publicly available [87]. In October 2011, The Cochrane Collaboration published a statement calling for all data from all randomised clinical trials, including raw anonymised individual participant data, to become publicly available free of charge and in easily accessible electronic formats [140].

## Competing interests

The author declares that he has no competing interests.
